# Efficacy and Safety of Ripretinib in Advanced Gastrointestinal Stromal Tumors within an Expanded Access Program: A Cohort Study

**DOI:** 10.3390/cancers16050985

**Published:** 2024-02-28

**Authors:** Su Yin Lim, Laura Ferro-López, Elizabeth Barquin, Daniel Lindsay, Khin Thway, Myles J. Smith, Charlotte Benson, Robin L. Jones, Andrea Napolitano

**Affiliations:** 1 The Royal Marsden NHS Foundation Trust, London SW3 6JJ, UK; suyin.lim1@nhs.net (S.Y.L.); laura.ferrolopez@rmh.nhs.uk (L.F.-L.); elizabeth.barquin@rmh.nhs.uk (E.B.); daniel.lindsay@rmh.nhs.uk (D.L.); khin.thway@rmh.nhs.uk (K.T.); charlotte.benson@rmh.nhs.uk (C.B.); robin.jones@rmh.nhs.uk (R.L.J.); 2 Institute of Cancer Research, London SW7 3RP, UK

**Keywords:** gastrointestinal stromal tumor, GIST, ripretinib, expanded access, real-world data

## Abstract

**Simple Summary:**

Ripretinib is a novel drug used to treat patients with advanced gastrointestinal stromal tumors. We investigated its efficacy and safety in a group of 45 patients treated in a real-world setting in the UK. We investigated the safety of the drug and its activity in causing tumor shrinkage and delaying tumor progression or death. Importantly, we also investigated the overall duration of the treatment, including when it was continued after radiological progression on the basis of clinical benefit, which is a common practice in the real-world setting. Our results show that both the efficacy and the safety of ripretinib in this group of patients were comparable to what had been reported in available clinical trials, supporting its use in patients with advanced gastrointestinal stromal tumors.

**Abstract:**

Ripretinib, a novel tyrosine kinase inhibitor used in advanced gastrointestinal stromal tumors (GIST) resistant to standard therapies, was assessed in the United Kingdom (UK) within an Expanded Access Program (EAP). A retrospective review of patients treated between January 2020 and October 2021 within the ripretinib EAP in our Institution was conducted. Clinician-documented and mRECIST 1.1 assessments were collected. The primary endpoints were progression-free survival (PFS) and time to treatment discontinuation (TTD). Treatment beyond progression (TBP), overall survival (OS), objective response rates and safety data were also analyzed. Survival curves were constructed using the Kaplan–Meier method, and univariate and multivariate Cox regression analyses were performed. All analyses were performed with R software. Overall, forty-five patients were included. After a median follow-up of 24.2 (95% CI 19.7–29.7) months, the median PFS of the group receiving 150 mg ripretinib once daily (OD) was 7.9 (95% CI 5.6–19.3) months. In the cohort of 22 patients with dose escalation upon tumor progression to 150 mg ripretinib twice daily (BD), the median PFS from BD was 5.4 (95% CI 2.8–9.3) months. Overall, median PFS and OS values for patients on ripretinib were 9.7 (95% CI 8.3–18.1) and 14.0 (95% CI 9.9–NA) months, respectively. TTD was similar to PFS. TBP was observed in about one third of all patients. Objective responses to ripretinib OD and BD treatments were observed in 16.7% and 10.0% of the patients, respectively. No new safety signals were identified. In conclusion, patients with advanced GIST receiving ripretinib in the UK within the EAP reported prolonged benefits, in line with the recent phase III clinical trials.

## 1. Introduction

Gastrointestinal stromal tumors (GIST) are rare mesenchymal malignancies of the gastrointestinal tract that are characterized by the frequent presence of oncogenic driver mutations in the KIT gene, which encodes the receptor tyrosine kinase c-KIT, and in the PDGFRA gene encoding the platelet-derived growth factor receptor α (PDGFRα) [1].

Targeted treatment of GIST with tyrosine kinase inhibitors (TKIs) has dramatically changed the outcome of the disease, with one in two patients now surviving for more than four years from the diagnosis of metastatic disease and one in five patients surviving for more than ten years [2].

Imatinib is the first-line TKI for most patients with KIT and PDGFRA mutations (including those with KIT exon 11 mutations, which are the most common); the multi-TKIs sunitinib and regorafenib are approved in the second- and third-line of treatment, respectively [3].

Ripretinib, a switch-control TKI targeting the c-KIT receptor through a novel mechanism of action, has been recently tested in two randomized phase III trials that demonstrated an excellent activity and safety profile [4]. In the INVICTUS trial, ripretinib significantly improved median progression-free survival (PFS) compared with placebo in patients with advanced GIST who were resistant to approved treatments (median PFS of 6.3 months with ripretinib compared with 1.0 months with placebo) [5]. In the INTRIGUE trial, ripretinib showed clinical activity comparable to second-line sunitinib, with improved tolerability [6].

The positive results of the INVICTUS trial led to the approval of ripretinib (Qinlock^®^) by the US Food and Drug Administration [7] and the European Medicines Agency [8] as a standard fourth-line treatment for patients with advanced GIST [3]. In the United Kingdom (UK), ripretinib is not currently approved for use.

Following the results of the INVICTUS trial, an international Expanded Access Program (EAP) was initiated to provide ripretinib to patients with disease progression following at least two prior lines of approved therapies (NCT04148092). The objective of this study was to describe the outcomes and safety of treatment with ripretinib in a large cohort of patients treated in our Institution as part of the UK EAP.

## 2. Materials and Methods

### 2.1. Study Design, Setting and Participants

A retrospective review of all patients with unresectable or metastatic GIST who commenced EAP ripretinib in our Institution (The Royal Marsden Hospital, London, UK) between January 2020 and October 2021 was performed. Patients without a confirmed histological diagnosis of GIST and patients who did not start ripretinib in our Institution (because they were too unwell or because they started in a different Institution) were excluded.

All patients treated in our Institution had imaging available for retrospective assessment of radiologic responses according to modified Response Evaluation Criteria in Solid Tumors (mRECIST) 1.1 and at least one measurable lesion. Radiological assessments were performed every 10–14 weeks or when clinically indicated. Retrospective mRECIST measurements were used to assess PFS, best objective response rate (ORR) and time to best response. Data cut-off was 15 January 2023.

### 2.2. Variables

Baseline patient characteristics included the following: sex; age in years at first ripretinib dose; primary mutational status (KIT and PDGFRA); primary tumor site (stomach, small bowel and other sites); stage (locally advanced or metastatic); number of metastatic sites (each organ counting as one site); Eastern Cooperative Oncology Group (ECOG) performance status (PS); and number and type of previous lines of treatment. ECOG PS was not used in subsequent univariate or multivariate analysis, as 42/45 (93.3%) of all patients were recorded as ECOG PS 1.

During treatment with ripretinib, information was collected on radiological response, toxicities (according to CTCAE v5), dose reductions, intra-patient dose escalation (IPDE) to 150 mg BD dosing, treatment duration and causes of treatment interruption. The same clinical and radiological information were also collected for patients treated with this higher dose. When documenting the reason for treatment discontinuation, we defined clinical progressive disease (PD) as worsening patient-reported symptoms in the presence of radiological signs of PD not amounting to mRECIST PD.

Given its observational nature within an EAP, this study had no predefined sample size, and all patients meeting the inclusion criteria were analyzed.

### 2.3. Outcomes

Due to the possibility of IPDE, we defined specific PFS endpoints: PFS OD was defined as the time from the date of the first 150 mg ripretinib OD dose to the time of mRECIST progression on this dose or death, whichever occurred first. PFS BD was defined, in the subgroup of patients undergoing IPDE, as the time from date of first 150 mg ripretinib BD dose to the time of mRECIST progression on this dose or death, whichever occurred first. To better capture the overall duration of ripretinib treatment regardless of the dosage, we also defined PFS intention-to-treat (ITT) as the time from the first 150 mg ripretinib OD dose to the time of mRECIST progression to this dose for those patients who did not undergo IPDE, and to the time of mRECIST progression to a 150 mg BD dose for those patients with dose escalation, or to time of death, whichever occurred first. Similarly, we defined time to treatment discontinuation (TTD) for the same groups as the time from their first day of ripretinib (at a specified dose) to their last day of ripretinib treatment (at a specified dose) regardless of the cause of treatment interruption. Treatment beyond progression (TBP) was defined as the time between the PFS event and the TTD event dates if the TTD event date was more than 28 days after the PFS event date. We defined overall survival (OS) as the time from the date of their first 150 mg ripretinib OD dose to the time of death for any cause.

Patients not experiencing the event were censored at the date of their last radiological evaluation for PFS endpoints, and to the date of their last clinic appointment for TTD and OS. Two patients with symptomatic and progressive disease were discharged to the local palliative care teams for the best supportive care and were then lost to follow-up. To avoid informative censoring, these patients were considered to have experienced the OS event 2 weeks after their last clinic appointment.

### 2.4. Statistical Analysis

Patient characteristics at baseline were reported as median (interquartile range, IQR) or percentages of the total population for continuous and categorical variables, respectively. Median follow-up time was estimated using the reverse Kaplan–Meier method. Survival curves were constructed using the Kaplan–Meier method, and univariate and multivariate Cox proportional hazard regression models were used to assess the prognostic value of baseline covariates. Variables with univariate *p* value < 0.2 were selected for the multivariate models. Hazard ratios (HR) along with their 95% confidence intervals (95% CI) were reported. The validity of the Cox proportional hazard assumption was tested for all multivariable models. No missing data were present. All analyses were performed with R software version 4.0.3 [9].

## 3. Results

### 3.1. Patient Selection, Baseline Characteristics and Disposition

In total, 61 patients were registered for the ripretinib EAP in our Institution. Of these, 16 patients were excluded (1 patient did not have GIST; 4 patients made their own decisions not to start ripretinib; 8 patients were too unwell to start ripretinib and 3 patients started the ripretinib EAP at a different Institution). Finally, 45 patients with available clinical and radiological data who were treated in our Institution were included.

Baseline patient characteristics are reported in Table 1. Information on primary mutational status was not available for six patients. In this study, the small bowel was the most common primary tumor site (51.1%); all patients except 1 had metastatic disease and a PS ECOG of 1 or 2; 26 (57.8%) patients had received 3 or more prior lines of treatment; all patients had progressed to imatinib and 95.6% of patients had also progressed to sunitinib. As it was allowed by the EAP, we also enrolled patients who only had received two prior lines of treatment to provide them with an additional line of treatment not otherwise available in the UK.

At the time of data cut-off, five (11.1%) patients were still receiving 150 mg ripretinib OD. Ripretinib OD was discontinued in the remaining 40 patients. Discontinuation was due to the following reasons: mRECIST PD in 21 (46.7%) cases, clinical PD in 17 (37.8%) cases, and toxicity in 2 (4.4%) cases. Of the 21 patients with mRECIST PD, 15 dose-escalated to ripretinib BD and 6 did not dose-escalate to ripretinib BD (2 received another tyrosine kinase inhibitor, and 4 declined IPDE and were referred back to local team). Of the 17 patients with clinical PD, 10 were too unwell to continue treatment and 7 dose-escalated to ripretinib BD. In total, 22 (48.9%) patients had their dose escalated to ripretinib BD.

Of the 22 patients undergoing IPDE, 4 (18.2%) were still receiving ripretinib BD at the time of data cut-off, 4 (18.2%) had clinical progression and 14 (63.6%) had mRECIST PD. Four patients went on to receive a further line of treatment after progression to ripretinib BD. Finally, at the time of data cut-off, 20 (44.4%) patients were still alive and 25 (55.6%) were dead.

### 3.2. Outcomes: Ripretinib OD

The median follow-up time in the whole population was 24.2 (95% CI 19.7–29.7) months. Three patients did not undergo radiologic evaluation, because of rapid clinical deterioration. In the 42 patients with at least one radiological evaluation, the best responses to ripretinib OD were partial response (PR) in 7 (16.7%) patients and stable disease (SD) in 29 (69.0%) patients. The median time to best response was 2.6 (IQR 1.9–3.3) months.

**Figure 1:**
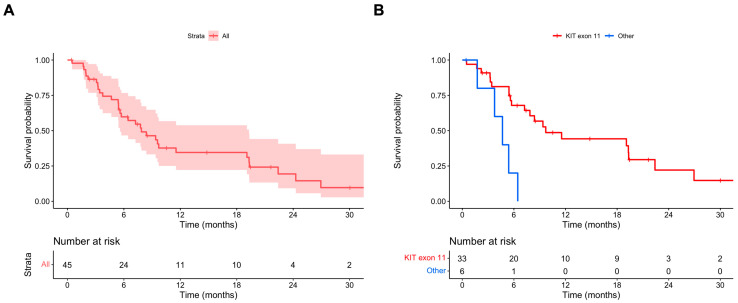
(**A**) PFS ripretinib OD; (**B**) PFS ripretinib OD stratified for primary mutational status (KIT exon 11 vs. other mutations).

The median PFS OD was 7.9 (95% CI 5.6–19.3) months (Figure 1A). In the univariate and multivariate Cox regression models, the absence of a KIT exon 11 mutation was associated with a statistically significant shorter PFS OD (multivariate HR 4.67, 95% CI 1.53–14.29) (Table 2). The median PFS OD values in patients with and without primary KIT exon 11 mutations were 9.7 (95% CI 7.2–26.9) and 4.7 (95% CI 95% CI 3.8–NA) months, respectively (Figure 1B).

The median TTD OD in the whole population was similar to the median PFS OD, being 7.1 (95% CI 5.8–11.0) months. In patients with and without KIT exon 11 mutations, the median TTD OD values were 8.5 (95% CI 5.9–15.0) and 3.5 (95% CI 2.7–NA) months, respectively. Ripretinib OD TBP was recorded in 14 (31.1%) patients. Patients who received TBP had no significant differences in baseline characteristics compared to patients who did not receive TBP, apart from site of disease (Supplementary Table S1). This difference is most likely due to the small sample size. The median duration of TBP was 1.9 months (IQR 1.0–3.0 months).

### 3.3. Outcomes: Ripretinib BD

Three patients did not undergo radiologic evaluation whilst on ripretinib BD; two because of rapid clinical deterioration and one because they were dose-escalated 2 weeks before data cut-off. In the 20 evaluable patients, the best responses to ripretinib BD were PR in 2 (10.0%) patients and SD in 9 (45.0%) patients.

The median PFS BD was 5.4 (95% CI 2.8–9.3) months (Supplementary Figure S1). Considering the smaller sample size, the Cox regression models were not conducted for this population. The median TTD BD was similar to the median PFS BD, being 6.5 (95% CI 4.6–10.7) months. Ripretinib BD TBP was observed in six (27.3%) patients. The median duration of TBP was 2.8 months (IQR 1.0–5.4 months).

### 3.4. Outcomes: Ripretinib ITT and OS

**Figure 2:**
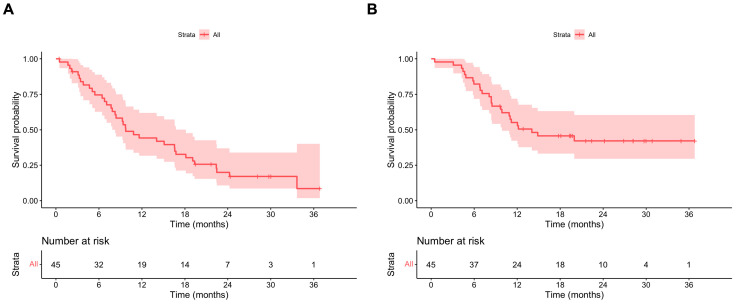
(**A**) PFS ripretinib ITT; (**B**) OS ripretinib ITT.

Overall, the median PFS ITT was 9.7 (95% CI 8.3–18.1) months (Figure 2A). The median PFS ITT values in patients with and without primary KIT exon 11 mutations were 14.0 (95% CI 8.4–19.3) and 6.4 (95% CI 95% CI 4.7–NA) months, respectively. In the multivariate model, the absence of a KIT exon 11 mutation was associated with a statistically significant shorter PFS ITT, with HR 3.06 (95% CI 1.08–8.67). Patients with three or more metastatic sites also had a statistically shorter PFS ITT compared to patients with less than two metastatic sites (HR 3.00, 95% CI 1.05–8.57) (Table 3).

The median OS for the whole population was 14.0 (95% CI 9.9–NA) months (Figure 2B). The median OS values in patients with and without primary KIT exon 11 mutations were 20.0 (95% CI 11.0–NA) and 7.2 (95% CI 95% CI 4.8–NA) months, respectively. In the multivariate model, the absence of a KIT exon 11 mutation was associated with a statistically significant shorter OS, with HR 4.19 (95% CI 1.49–11.82) (Supplementary Table S2).

### 3.5. Toxicity

The toxicity profile observed was in line with available data from randomized studies in the literature [5,6], with most toxicities being CTCAE grade (G) 1 or 2. The most common toxicities reported in our cohort were fatigue, alopecia, palmar-plantar erythrodysesthesia syndrome (PPE) and gastrointestinal and musculoskeletal symptoms (Table 4).

During treatment with 150 mg ripretinib OD, ten (22.2%) patients required dose reductions. Two (4.4%) patients discontinued ripretinib OD due to persistent toxicities despite adequate dose reductions (one patient due to G2 PPE associated with other G1 toxicities, and one patient due to G2 fatigue and G2 constipation associated with other G1 toxicities). After IPDE, 2 (8.7%) patients required dose reductions. No patient discontinued ripretinib BD due to toxicities. No treatment-related deaths occurred.

## 4. Discussion

This study investigated the outcomes of advanced GIST patients treated within the ripretinib EAP in our Institution in the UK. We confirmed the significant clinical efficacy of ripretinib in pre-treated GIST patients: the observed objective response rates, median PFS and median OS are entirely consistent with those reported in the phase III INVICTUS [5] and INTRIGUE [6] trials, allowing for the main differences between trials and the real-world setting in the frequency of radiologic assessments, percentage of patients with baseline ECOG PS 1, and TBP.

The continuation of treatment with TKIs beyond evidence of radiologic progression is a common event in clinical practice across multiple oncogene-driven cancer types. This approach is specifically supported in imatinib-resistant GIST by the evidence of a rapid deterioration of patients treated with placebo within clinical trials [10]. Moreover, the clinical value of a continued inhibition of c-KIT, though sub-optimal, is further supported by the benefit reported after imatinib rechallenge in patients who have exhausted all available treatment options [11,12]. Importantly, real-world data represent a unique source of information to study the frequency and duration of TBP, as its use—though relatively limited in our study—could affect national approval and reimbursement strategies.

In the dose-escalation phase of the ripretinib phase I study (NCT02571036), the maximum tolerated dose was not reached among the doses tested. For this reason, ripretinib IPDE to 150 mg BD was tested during the phase I study with meaningful outcomes in patients progressing to 150 mg OD [13]. This was allowed in both the INVICTUS study [14] and in the EAP. Our analysis confirms these positive results, with prolonged clinical and radiological benefit in patients who underwent IPDE.

Whilst IPDE would confound the potential survival outcome analyses if ripretinib were only given at the labelled dose of 150 mg OD, our results might help with modelling them. In fact, the fraction of patients who were offered IPDE in the EAP (~50% of the total patients) would be likely in clinical practice to receive TBP at the 150 mg OD dose for a median duration of ~2 to 3 additional months.

It is worth noting that tumor response was assessed both in the INTRIGUE and INVICTUS clinical trials using mRECIST 1.1, and we therefore retrospectively employed the same radiologic criteria. However, the Choi response criteria might be more sensitive and precise in assessing the responses of GISTs to TKIs [15], and it will be important in the future to ascertain whether they might help with assessing responses to ripretinib as well.

Notably, in our cohort there was no association between the number of previous lines of treatment and survival outcomes while on ripretinib. This suggests that the actual mutational status might be more important in determining an individual patient’s sensitivity to the treatment than the number of previous treatments received. Unfortunately, the lack of tissue samples collected immediately prior to starting ripretinib limits our capability to correlate a tumor’s intrinsic (e.g., mutational status) and extrinsic characteristics (e.g., immune microenvironment) with its response to ripretinib. In our analyses, the presence of a primary KIT exon 11 mutation was consistently associated with significantly longer PFS, TTD and OS, confirming its prognostic value. In the phase III INVICTUS trial, ripretinib showed efficacy over placebo across different mutational subgroups [16], though there is initial evidence that the mutational status might be a predictor of response to ripretinib in earlier lines of treatment [6,17], when the mutational heterogeneity is lower. In patients with advanced disease and multiple metastatic sites, future longitudinal studies would benefit from the analysis of circulating tumor DNA (ctDNA) to better characterize the subpopulations deriving the greatest benefit from treatment with ripretinib [17]. Patient selection with ctDNA is currently being implemented in the INSIGHT phase III clinical trial comparing ripretinib to sunitinib in the second-line setting within patients belonging to a specific mutational subgroup, i.e., patients with mutations in KIT exon 11 + KIT exon 17/18 only (NCT05734105).

Our study is limited by its relatively small size, which might not be completely representative of the advanced GIST patient population with regards to disease origin and mutational status. In fact, in our cohort we observed an unusual prevalence of small bowel GIST. Though it is difficult to explain this finding, this is unlikely to have had a major impact on our results, as the site of origin was not associated with any outcome in our survival models.

Ripretinib is generally well tolerated based on both recorded toxicities [5,6] and, importantly, on patient-reported quality-of-life questionnaires [18,19]. Interestingly, toxicities of grade 3 or higher were less frequent in the BD group. As only patients tolerating ripretinib OD well were eligible for IPDE, there could be a selection bias in the BD group. Importantly, given the retrospective and observational nature of our study, it is likely that our adverse events might be under-reported and formal assessment of patient-reported outcomes was not possible. However, our data did not highlight any new safety signals, and the reported good tolerability of ripretinib was confirmed overall.

## 5. Conclusions

Our study supports the role of EAPs as a means to gather early real-world evidence on the safety and efficacy of drugs, including ripretinib [20,21], outside of clinical trials. It confirms treatment with ripretinib as an effective strategy for patients with advanced GIST in the UK.

## Figures and Tables

**Table 1:** Baseline patient characteristics.

Total Number of Patients	45	100%
Sex		
Female	19	42.2%
Male	26	57.8%
Age		
Median, Q1–Q3	62	57–72
Primary mutational status		
KIT exon 11	33	73.3%
KIT exon 9	3	6.7%
PDGFRA	3	6.7%
N/A	6	13.3%
Primary tumor site		
Stomach	13	28.9%
Small bowel	23	51.1%
Other	9	20.0%
Stage		
Locally advanced	1	2.2%
Metastatic	44	97.8%
1 site	15	34.1%
2 sites	18	40.9%
3 or more sites	11	25.0%
ECOG		
0	1	2.2%
1	42	93.3%
2	2	4.4%
Number of previous lines		
2	19	42.2%
3 or more	26	57.8%
Previous lines		
Imatinib	45	100%
Sunitinib	43	95.6%
Regorafenib	24	53.3%
Avapritinib	13	28.9%

N/A: not available.

**Table 2:** Univariate and multivariate Cox regression models for PFS OD.

Variable	Univariate HR (95% CI)	*p* Value	Multivariate HR (95% CI)	*p* Value
Sex (male vs. female)	1.54 (0.75–3.15)	0.238	NI ^1^	
Age	1.00 (0.97–1.03)	0.923	NI	
Primary mutation (others vs. KIT exon 11)	4.98 (1.65–15.03)	0.004 *	4.67 (1.53–14.29)	0.007 *
Primary tumor site			NI	
Small bowel vs. gastric	0.96 (0.41–2.22)	0.918		
Others vs. gastric	0.98 (0.36–2.65)	0.862		
Number of metastatic sites				
2 vs. 0/1	1.79 (0.76–4.23)	0.185	1.59 (0.61–4.14)	0.342
3 or more vs. 0/1	2.28 (0.86–6.02)	0.098	2.19 (0.72–6.71)	0.170
Number of previous lines (3 or more vs. 2)	0.88 (0.43–1.77)	0.711	NI	
Previous regorafenib	1.09 (0.53–2.21)	0.919	NI	
Previous avapritinib	1.27 (0.59–2.77)	0.578	NI	

^1^ NI: not included. *: statistically significant.

**Table 3:** Univariate and multivariate Cox regression models for PFS ITT.

Variable	Univariate HR (95% CI)	*p* Value	Multivariate HR (95% CI)	*p* Value
Sex (male vs. female)	1.46 (0.75–2.84)	0.263	NI ^1^	
Age	1.01 (0.98–1.04)	0.397	NI	
Primary mutation (others vs. KIT exon 11)	2.75 (1.00–7.58)	0.051	3.06 (1.08–8.67)	0.036 *
Primary tumor site			NI	
Small bowel vs. gastric	1.20 (0.55–2.62)	0.641		
Others vs. gastric	0.78 (0.29–2.08)	0.616		
Number of metastatic sites				
2 vs. 0/1	2.01 (0.88–4.57)	0.096	1.94 (0.78–4.78)	0.153
3 or more vs. 0/1	2.41 (0.97–6.02)	0.059	3.00 (1.05–8.57)	0.040 *
Number of previous lines (3 or more vs. 2)	0.85 (0.44–1.67)	0.645	NI	
Previous regorafenib	0.85 (0.44–1.64)	0.623	NI	
Previous avapritinib	1.15 (0.54–2.47)	0.714	NI	

^1^ NI: not included. *: statistically significant.

**Table 4:** Toxicities reported in at least 20% of patients.

	Ripretinib OD (N = 45)		Ripretinib BD (N = 23)	
Adverse Event	Any G (N, %)	G3+ (N, %)	Any G (N, %)	G3+ (N, %)
Fatigue	35 (77.8%)	0 (0.0%)	18 (78.3%)	0 (0.0%)
Alopecia	21 (46.7%)	0 (0.0%)	9 (39.1%)	0 (0.0%)
Muscle cramp	20 (44.4%)	0 (0.0%)	4 (17.4%)	0 (0.0%)
PPE	17 (37.8%)	1 (2.2%)	9 (39.1%)	0 (0.0%)
Constipation	17 (37.8%)	1 (2.2%)	8 (34.8%)	0 (0.0%)
Arthralgia/myalgia	15 (33.3%)	1 (2.2%)	6 (26.1%)	0 (0.0%)
Weight loss	14 (31.1%)	0 (0.0%)	13 (56.5%)	0 (0.0%)
Diarrhea	13 (28.9%)	2 (4.4%)	15 (65.2%)	1 (4.4%)
Anorexia	10 (22.2%)	0 (0.0%)	8 (34.8%)	0 (0.0%)

## Data Availability

Data are available upon request to the corresponding author with a proposal for their use. Data transfer is subject to Institutional agreements.

## Supplementary Materials

The following supporting information can be downloaded at https://www.mdpi.com/article/10.3390/cancers16050985/s1: Figure S1, PFS ripretinib BD; Table S1, Differences in baseline patient characteristics stratified by ripretinib OD TBP; and Table S2, Univariate and multivariate Cox regression model for OS.
