# Supplementary material for: Efficacy and Safety of Ripretinib in Advanced Gastrointestinal Stromal Tumors within an Expanded Access Program: A Cohort Study
Source: Cancers (Basel). 2024 Feb 28;16(5):985. doi: 10.3390/cancers16050985 (PMC10930637; doi:10.3390/cancers16050985)
Supplement: Supplementary file 1 [file cancers-16-00985-s001.zip › cancers-2853339-supplementary.pdf]

Efficacy and Safety of Ripretinib in Advanced Gastrointestinal Stromal Tumors within an Expanded Access Program: A Cohort Study

Su Yin Lim [1], Laura Ferro Lopez [1], Elizabeth Barquin [1], Daniel Lindsay [1], Khin Thway [1,2], Myles J Smith [1,2], Charlotte Benson [1], Robin L Jones [1,2] and Andrea Napolitano [1]

1. The Royal Marsden NHS Foundation Trust, London SW3 6JJ, United Kingdom
2. The Institute of Cancer Research, London SW7 3RP, United Kingdom

- **Supplementary Figure S1.** PFS ripretinib BD
- **Supplementary Table S1.** Differences in baseline patient characteristics stratified by ripretinib OD TBP
- **Supplementary Table S2.** Univariate and multivariate Cox regression model for OS

Supplementary Figure S1. PFS ripretinib BD

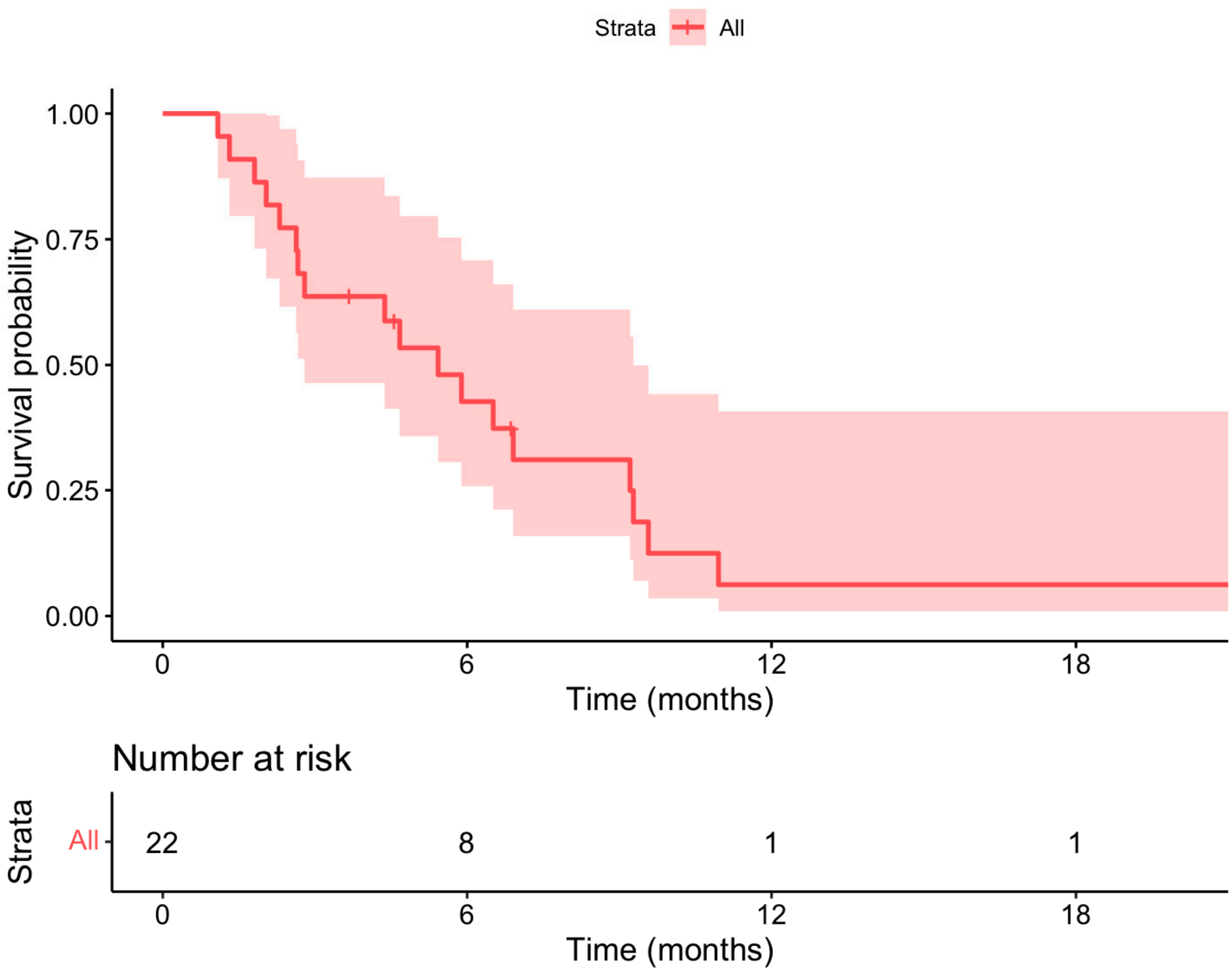

Supplementary Table S1. Differences in baseline patient characteristics stratified by ripretinib OD TBP

**Ripretinib OD TBP**	**No**	**Yes**	***P* value**
**Number of patients**	31	14	
**Sex:** Male	19 (61.3%)	7 (50%)	0.701
**Age** (mean, standard deviation)	63 (10)	64 (15)	0.672
**Primary mutational status**			0.569
- *KIT* exon 11	24 (77.4%)	9 (64.3%)	
- *KIT* exon 9	2 (6.5%)	1 (7.1%)	
- *PDGFRA*	1 (3.2%)	2 (14.3%)	
- N/A	4 (12.9%)	2 (14.3%)	
**Primary tumour site**			0.026
- Stomach	9 (29.0%)	4 (28.6%)	
- Small bowel	19 (63.3%)	4 (28.6%)	
- Other	3 (9.7%)	6 (42.9%)	
**Stage:** metastatic	31 (100.0%)	13 (92.9%)	0.680
**Number of metastatic sites**			0.342
- 1 site	9 (29.0%)	7 (50%)	
- 2 sites	13 (41.9%)	5 (35.7%)	
- 3 or more sites	9 (29.0%)	2 (14.3%)	
**ECOG**			0.484
- 0	1 (3.2%)	0 (0.0%)	
- 1	28 (90.3%)	14 (100.0%)	
- 2	2 (6.5%)	0 (0.0%)	
**Number of previous lines:** 3 or more	20 (64.5%)	6 (42.9%)	0.300
**Previous lines**			
- Sunitinib	30 (96.8%)	13 (92.9%)	1.000
- Regorafenib	19 (61.3%)	5 (35.7%)	0.204
- Avapritinib	9 (29.0%)	4 (28.6%)	1.000

Supplementary Table S2. Univariate and multivariate Cox regression model for OS.

**Variable**	**Univariate HR (95% CI)**	***P* value**	**Multivariate HR (95% CI)**	***P* value**
Sex (male vs female)	2.45 (1.02–5.91)	0.045 *	1.70 (0.66–4.38)	0.272
Age	1.00 (0.97–1.03)	0.951	NI	
Primary mutation (others vs KIT exon 11)	3.39 (1.30–8.82)	0.012 *	4.19 (1.49–11.82) *	0.007 *
Primary tumour site			NI	
- Small bowel vs gastric	1.08 (0.45–2.60)	0.872		
- Others vs gastric	0.70 (0.21–2.34)	0.566		
Number of metastatic sites				
- 2 vs 0/1	1.44 (0.55–3.78)	0.462	1.01 (0.35–2.97)	0.981
- 3 or more vs 0/1	2.16 (0.78–5.96)	0.139	2.37 (0.74–7.56)	0.145
Number of previous lines (3 or more vs 2)	0.98 (0.44–2.19)	0.961	NI	
Previous regorafenib	1.04 (0.51–2.11)	0.919	NI	
Previous avapritinib	1.25 (0.57–2.72)	0.578	NI	

NI: not included
